# Low Dose Rapamycin Alleviates Clinical Symptoms of Fatigue and PEM in ME/CFS Patients via Improvement of Autophagy

**DOI:** 10.21203/rs.3.rs-6596158/v1

**Published:** 2025-06-03

**Authors:** Brian T. Ruan, Sarojini Bulbule, Amy Reyes, Bela Chheda, Lucinda Bateman, Jennifer Bell, Braydon Yellman, Stephanie Grach, Jon Berner, Daniel L. Peterson, David Kaufman, AVIK ROY, C. Gunnar Gottschalk

**Affiliations:** Cornell University; Simmaron Research INC; University of Wisconsin-Milwaukee; Center for Complex Diseases, Palo Alto, CA; Bateman Horne Center; Bateman Horne Center; Bateman Horne Center; Mayo Clinic Research Rochester; Woodinville psychiatric Associates; Sierra Internal Medicine; Center for Complex Diseases; University of Wisconsin-Milwaukee; Simmaron Research INC

## Abstract

**Background:**

mTOR activation is associated with chronic inflammation in ME/CFS. Previous studies have shown that sustained mTOR activation can cause chronic muscle fatigue by inhibiting ATG13-mediated autophagy. This highlights the pivotal role of mTOR in the pathogenesis of ME/CFS.

**Methods:**

We conducted a decentralized, uncontrolled trial of rapamycin in **86** patients with ME/CFS to evaluate its safety and efficacy. Low-dose rapamycin (6 mg/week) was administered, and core ME/CFS symptoms were assessed on days 30 (T1), 60 (T2), and 90 (T3). Plasma levels of autophagy metabolites, such as pSer258-ATG13 and BECLIN-1, were measured and correlated with clinical outcomes, specifically MFI.

**Results:**

Rapamycin (6 mg/week) was tolerated without any SAEs. Of the 40 patients, 29 (72.5%) showed strong recovery in PEM, fatigue, and OI, along with improvements in MFI fatigue domains and SF-36 aspects. High levels of BECLIN-1 were detected in T3. Plasma pSer258-ATG13 levels were strongly downregulated at T1. Spearman’s correlation analysis indicated an association between autophagy impairment and reduced activity.

**Conclusions:**

Low-dose rapamycin effectively reduced PEM and other key symptoms in patients with ME/CFS, as measured by BAS, SSS, MFI, and SF-36. Future studies should encompass dose optimization and develop a diagnostic tool to identify responders with mTOR-mediated autophagy disruption.

## Introduction

Myalgic encephalomyelitis/chronic fatigue syndrome (ME/CFS) is a complex, multisystem disorder characterized by profound fatigue, post-exertional malaise (PEM), musculoskeletal pain, cognitive dysfunction, and orthostatic intolerance (OI)^1,2^. An estimated 3 million individuals in the United States are affected by ME/CFS^3^. Diagnostic criteria established by the Institute of Medicine, the 2003 Canadian Consensus Criteria, and the Fukuda Criteria (endorsed by the CDC) all emphasize PEM as the cardinal feature distinguishing ME/CFS from other clinically similar conditions^4,5^. PEM is defined as a delayed and prolonged exacerbation of ME/CFS symptoms, including severe fatigue, myalgia, light and sound sensitivity, flu-like symptoms, and cognitive dysfunction following minimal physical or mental exertion. These episodes are often incapacitating, typically lasting more than 72 h, and in some cases persisting for weeks or longer. Evidence suggests that recurrent or overlapping PEM episodes may be additive and potentially contribute to irreversible physiological deterioration, underscoring the need for effective therapeutic intervention. OI, another hallmark of ME/CFS^6^, is characterized by abnormal cardiovascular responses to an upright posture, including hypotension and tachycardia. Despite substantial research efforts, the pathophysiology of ME/CFS remains unclear. Emerging evidence implicates abnormalities in several metabolic and cellular pathways, including impaired glycolysis^7,8^, reduced mitochondrial oxygen consumption^9^, deficits in energy metabolism^10^, and disrupted autophagy^11^ as potential contributors to disease pathogenesis.

Currently, no FDA-approved therapies are available for the treatment of ME/CFS. Symptomatic treatments—used off-label to manage key features such as post-exertional malaise (PEM) and orthostatic intolerance (OI)—offer limited benefit and lack a well-defined mechanism of action. The absence of mechanistic clarity makes it challenging to predict therapeutic responses in a highly heterogeneous patient population.

Our previous study^11^ demonstrated that serum samples from ME/CFS subjects exhibited elevated levels of the early autophagy protein, ATG13. Further analysis revealed that the elevated ATG13 in these serum samples was heavily phosphorylated at its serine residue, rendering it non-functional and potentially indicative of impaired autophagy. Accordingly, in a mouse model^12^, genetic ablation of ATG13 resulted in severe muscle fatigue following treadmill exercise, suggesting a crucial role for ATG13 in the pathogenesis of ME/CFS. The mammalian target of rapamycin (mTOR) is the upstream kinase^13^ responsible for phosphorylating ATG13^14^ at its Ser258 residue^15^ during starvation-induced autophagy impairment. Notably, our study^12^ found that the pharmacological activation of mTOR through oral administration of the mTOR agonist MHY1485 resulted in serine phosphorylation at ATG13 and impaired macroautophagy by dislodging ATG13 from the early autophagy complex, leading to mononucleosis and increased infiltration of inflammatory macrophages in the vasculature of muscle tissue, which caused demyelination of nerves serving muscles. Other studies^16,17^ have also reported that the activation of mTOR in monocytes induces pro-inflammatory responses in chronic cardiovascular diseases. These studies strongly suggest that inactivation of mTOR may be beneficial in alleviating the pathological symptoms of chronic inflammatory diseases. To investigate the role of mTOR inhibition in alleviating clinical symptoms of ME/CFS, we initiated an observational trial with low-dose, once weekly (6 mg/wk) Rapamycin (*sirolimus*), an mTOR inhibitor, in ME/CFS subjects.

Rapamycin, a selective mTOR inhibitor^18,19^, was approved by the FDA in 1999^20^ as an immunosuppressant to prevent organ rejection and later in 2003 for use in cardiac stents to prevent restenosis^21^. It is actively researched and used off-label for various cancers^22,23^, including tuberous sclerosis^24^, acute myeloid leukemia^25^, and cancers of renal^26^, breast^27^, and lung^28^ origins. Its safety profile has been well studied^29^. Rapamycin has been recently investigated as a geroprotective drug^30^. A study conducted^30^ by the Interventions Testing Program (ITP), a peer-reviewed NIH program, along with other research^31^, has led to ongoing trials of rapamycin for longevity in humans^33^ as well as its widespread off-label use for geroprotection^32^.

Low-dose rapamycin (6 mg/week) was administered to 86 individuals with ME/CFS, of whom 40 completed the full 90-day study protocol. As of the submission of this manuscript, 6 patients remain on the study protocol. The study included four time points for clinical and biospecimen data collection: a pretreatment baseline (BSL; day 0), followed by longitudinal assessments at day 36 (T1), day 60 (T2), and day 90 (T3), while participants remained on therapy. At each time point, peripheral blood was collected via mobile phlebotomy, and samples were processed and analyzed at the Simmaron-UWM Laboratory. Safety laboratory assessments were performed concurrently using LabCorp^®^. Clinical endpoints were assessed using self-reported validated instruments endorsed by the NIH Common Data Elements for ME/CFS, including measures of fatigue, post-exertional malaise (PEM), orthostatic intolerance (OI), and related symptoms. Biological assays focused on quantifying autophagy-associated proteins, including BECLIN-1 and phosphorylated ATG13 at serine 258 (pSer258-ATG13), at each time point. Nonparametric correlation analyses demonstrated significant clinical improvement in 38 of the 40 participants who completed the study, characterized by reductions in fatigue, PEM, and OI. These clinical responses were accompanied by the upregulation of BECLIN-1 and suppression of pSer258-ATG13, supporting a mechanistic link between the therapeutic benefit and restoration of autophagy signaling.

## Materials and methods

### Trial Design, IRB approval, and Oversight:

This rapamycin trial in ME/CFS was a non-controlled, decentralized, observational clinical study conducted across six clinical centers in the United States. Participants were recruited and remained under the care of site-specific principal investigators at the following institutions: The Center for Complex Diseases (Bela Chheda, MD, Mountain View, CA, and David Kaufman, MD, Seattle, WA), the Bateman Horne Center (Jennifer Bell, FNP, Salt Lake City, UT), Simmaron Research Clinical Center (Daniel Peterson, MD, Incline Village, NV), Mayo Clinic (Stephanie Grach, MD, Rochester, MN), and Woodinville Psychiatric (Jon Berner, MD, PhD, Woodinville, WA). The study was conducted under a central protocol (SRI-RP-2023–1) and all sites were approved by the Western Institutional Review Board (WIRB Study Number: 1361805). Simmaron Research Inc. is the study sponsor and the trial was registered on ClinicalTrials.gov (NCT06257420).

### Selection of patient cohort and other general information:

Participants (N=109) were enrolled in this trial by invitation only. Each participant was pre-screened by the site principal investigators to confirm that they met the clinical diagnostic criteria for ME/CFS based on the IOM criteria. Written informed consent was obtained from all participants or their legal guardians prior to enrollment. Once enrolled, participants were continuously monitored by physicians at each clinical site.

### Safety Lab and Study Sample Collection:

Upon completion of the baseline questionnaires, a certified mobile phlebotomy provider was scheduled for each enrolled participant. Four study sample kits (BSL-T3) were shipped to the participants’ residences prior to the scheduled blood collection. Each kit contained the following BD Vacutainer^®^ tubes (Franklin, NJ, USA): two 10 mL K2EDTA tubes, one 10 mL SST tube, one 4 mL sodium citrate (NaCit) tube, and one RNA PaxGene tube. Concurrent with the study sample collection, safety laboratory tests were performed using the same phlebotomy visit and submitted to LabCorp^®^ for analysis. Safety labs included the following assays: Comprehensive Metabolic Panel (CMP-14, #322000), Complete Blood Count with Differential (CBC-Diff, #005009), Lipid Panel (#303756), High-Sensitivity C-Reactive Protein (HS-CRP, #120766), and Hemoglobin A1C (#001453). Ordering physicians monitored the results. All samples were processed under Good Laboratory Practice (GLP) conditions within 12 h of collection at the Simmaron Research and Development Laboratory (University of Wisconsin–Milwaukee). Plasma, serum, and PaxGene RNA were extracted from whole blood, aliquoted immediately, and stored at − 80°C or in liquid nitrogen. Demographic and clinical data including age, sex, ethnicity, body mass index (BMI), and disease duration were recorded at enrollment and at each time point thereafter.

### Digital database maintenance in REDCap:

The REDCap database used in this study was approved, housed, and managed at the Weill Cornell School of Medicine REDCap server in collaboration with the Cornell EIND Center^33, 34^. Electronic versions of all consent forms and study questionnaires were housed in the REDCap database.

### Study questionnaires (BSL-T3):

After resigning the electronic site-specific informed consent document, the patient completed the BSL questionnaires (electronically), which included the Medical Outcomes Study-36 Item Short Form-Health Survey (SF-36), Multidimensional Fatigue Inventory (MFI), Bell Activity Scale (BAS), Sleep Questionnaire, Specific Symptom Severity (SSS) Inventory, Medication List, Past and Current Illnesses (BSL only), Questionnaires for ME/CFS Patients (BSL only), and the 24-Hour-Health-Inventory. Follow-up questionnaires were distributed via REDCap at each time point, in line with the study protocol.

### The measurement protocol of the BAS, SSS, MFI, and SF-36:

All participants were asked to complete a series of validated questionnaires and surveys to gauge the complexity and severity of symptoms over time. The primary study endpoints included BAS, SSS, MFI, and SF-36 scores. The BAS ranges from 0 to 100, where 0 indicates severe symptoms on a continuous basis, and 100 indicates no symptoms at rest or with exercise^35^. Participants also completed the SSS instrument, reporting the severity of common ME/CFS symptoms on a scale of 0 to 10, where 0 indicates not experienced and 10 indicates very severe symptoms. This inventory includes four hallmark symptoms of ME/CFS: fatigue, disturbed sleep, post-exertional malaise, and orthostatic intolerance. The MFI consists of 20 questions on a scale of 1 to 5, quantifying fatigue in five different domains^36^. Each domain consists of four items with scores between 4 and 20, where 4 indicates low to no fatigue and 20 indicates severe fatigue. The patients also completed the RAND 36-Item Short Form Health Survey (SF-36, version 1), which quantifies various quality of life measures^37^. Scores were scaled to the mean of the healthy US population according to the scoring instructions^38^.

### Stratification based on BAS, MFI, and SF-36 criteria for responder analysis:

The current literature is limited compared to responder analysis in ME/CFS clinical trials. However, previous studies^39–41^ have examined empirical methods to identify patients with ME/CFS using the SF-36. The most relevant subscales were VT, SF, and RP. Another study^42^ included the PCS aggregate score in their responder analysis of patients with anemia and chronic kidney disease. They also identified a three-point differential in the SF-36 subscales and a two-point differential in the PCS as the threshold for a minimal clinically important difference (MCID) between time points. Accordingly, the ME/CFS patients in this rapamycin trial were stratified as follows.

#### SF-36

· Responder: Two or more of the following:
  o ≥ +3 in VT sub-scale
  o ≥ +3 in SF sub-scale
  o ≥ +3 in RP sub-scale
  o ≥ +2 in PCS sub-scale
· Partial responder: One or more of the above
· Non-responder: None of the above

Stroke rehabilitation studies^43^ have proposed a −5 to −7.33-point differential as the MCID for the MFI aggregate score. For ME/CFS, a minus two-point differential was established as the MCID for each MFI fatigue domain^44^. An extrapolation to the MFI aggregate would suggest that a minus-ten-point differential is required as the MCID for the MFI aggregate score. Accordingly, participants were stratified as follows:

#### MFI

· Responder:
  o ≤ −2 for three or more of the five fatigue domains
  o ≤ −2 in two of the five domains AND ≤ −10 in the MFI aggregate
· Partial responder:
  o ≤ −2 for two or more of the five fatigue domains.
  o ≤ −2 in one of the five domains and −10 < MFI aggregate < 0.
· Non-responder: None of the above

Although the BAS is frequently employed in ME/CFS studies^45^ to quantify functional ability, its use as a formal responder classification tool remains limited. Thus, we propose a new methodology for future responder analysis.

#### BAS

· Responder: ≥ +10 in BAS
· Non-responder: ≤ 0 in BAS

The partial responder stratum was excluded because the BAS operates at intervals of ten, and a ten-point differential in the BAS is descriptively significant. Furthermore, a theoretical twenty-point differential for the responder subset to accommodate a partial responder subset would be too conservative for a stratification approach.

Finally, to classify participants, their overall responder status was determined as such:

· Responder: ≥ 2 “responders” in SF-36, MFI, or BAS
· Partial responder: ≥ 1 < BA > < BA > “partial responder” in SF-36, MFI, or BAS
· Non-responder: None of the above

Differentials were measured from baseline to the last completed timepoint for each participant.

### Training a machine learning model to subset participants using random forest classifier:

Data were first preprocessed to include only the features of interest: BAS, MFI (all), SF-36 RP, VT, SF subscales, and the PCS aggregate score. Because there were missing data, k-nearest neighbor (KNN) imputation was used to impute these missing values. The data were then split with a test size of 0.20 for training and testing. After the model was trained using the KNN-imputed data, hyperparameter tuning using GridSearch with 5-fold cross-validation was performed to determine the optimal parameters for our random forest. A confusion matrix and feature importance analysis were completed, and then the area under the curve (AUC) receiver operator curve (ROC) was graphed. The random forest workflow required the use of Python version 3.12.8 with the scikit-learn package version 1.6.1.

### Study events:

Safety laboratory assessments, study sample collection, and RedCap questionnaire administration were conducted according to a standardized study timeline. Assessments were performed at the following time points: baseline (within two weeks prior to initiating rapamycin treatment), time point 1 (T1; following a six-week dose escalation from 1 mg/week to 6 mg/week), time point 2 (T2; 30 days after T1), and time point 3 (T3; 30 days after T2). Additional optional follow-up assessments (T3–T7) were conducted every 90 days following T3 using the same schedule of events. Upon initiation of the first rapamycin dose, the study team scheduled subsequent study events to ensure adherence to the protocol timeline.

### Clinical monitoring:

All participants completed regular telemedicine and/or in-person office visits with their enrolling clinician (clinical PI). Patients communicated any AEs, SAEs, dose changes, or dose stoppages directly to their clinicians and to the overall study PI. These findings have been reported and documented previously.

### Obtaining study drug:

After reviewing the baseline safety laboratories and monitoring drug-drug interactions, study physicians prescribed the study drug, which the patients filled at their local pharmacy of choice.

### Biobanking of serum, plasma, and PBMCs:

Upon receipt, plasma samples were aliquoted and immediately stored in a −80°C freezer located at suite #353 of the Milwaukee Institute for Drug Discovery (MIDD). PBMCs were isolated using an optimized magnetic separation protocol developed by Stem Cell Technologies (Cambridge, MA), counted using an automated digital counter (Invitrogen Countess, Invitrogen, Waltham, MA), and then stored in a liquid nitrogen storage tank (ThermoFisher INC) at MIDD.

### ELISA analysis of BECLIN-1:

ELISA of BECLIN-1 was performed based on a kit (Cat# ab254511) and by a protocol described in the manufacturer’s protocol

### Development of ATG13 ELISA and analysis in plasma samples.

The Ser258 phospho-specific ATG13 antibody was developed for this study in collaboration with Thermo Fisher Scientific’s custom antibody synthesis division. The standard immunization protocol was adopted by injecting peptide antigen (0.5 mg) into rabbits and collecting serum samples 28, 56, and 72 days after the first immunization. The crude sera were affinity-purified, validated by ELISA with serial dilution, and provided to our laboratory. For ELISA, Nunc MicroWell 96-Well Optical-Bottom Plates with polystyrene Base (Cat# 165305; ThermoFisher) were used. Briefly, the plasma samples were mixed with carbonate coating buffer (Cat# CB01100; ThermoFisher Scientific) at a dilution of 1:2 (v/v), followed by addition to the plate overnight at 4 °C. After the coating process, the plate was blocked with 2% goat serum (Cat# 01–6201; ThermoFisher) for 30 min at room temperature and then treated with our custom-made pSer258-ATG13 antibody at a dilution of 1:100 with TBS-based antibody diluent (Li-cor Biosciences). After 2 h of incubation, the plate was washed with 1X TBST for three times followed by incubation with biotin-conjugated goat anti-rabbit IgG (H+L) (Cat# A16100) for 1 h. After that, 3X washes with 1XTBST were performed, streptavidin-HRP conjugate (Cat# S911; ThermoFisher) was added for 30 min, and the color was finally developed with the TMB substrate solution. The color development was stopped by 0.1N hydrochloric acid (stop solution) before saturation.

### Statistical analysis:

Demographic data were collected and are summarized in Figure 3A, stratified by history of viral onset in Figure 7A, and by responder status in Table 5A. The mean, range, and p-values from Student’s t-test were calculated for continuous variables. Percentages and p-values from the chi-square test of independence were calculated for categorical variables. Participant summary characteristics were prepared using pandas version 2.2.3 and SciPy version 1.15.1, and Python version 3.11.11.

The Shapiro-Wilk test of non-normality was performed for each time point. (See Supplemental Statistical Analysis for the results). Variations over time were plotted using column graphs with individual points and SEM bars. Serum concentrations of BECLIN-1 and pSer258-ATG13 were log2-transformed. Repeated measures ANOVA (RM ANOVA) was used to identify statistically significant differences between time points for BECLIN-1. The questionnaire and pSer258-ATG13 data were handled differently because some participants did not complete the questionnaires. A mixed effects model was thus fit using restricted maximum likelihood (REML) to produce p-values, similar to an RM ANOVA. For subset analyses, two-way ANOVA (or a matched mixed-effects model for data with missing values) was used to further evaluate any interaction effects between time points and subsets. Geisser-Greenhouse correction was used to correct for the assumption of sphericity in all variance analyses. Simple effects within participants comparing each time point to the baseline were corrected for multiple comparisons using Dunnett’s method. QQ plots were plotted for each ANOVA or mixed-effect model to test the normality of the residuals. Bivariate analysis was conducted using Spearman’s correlation and simple linear regression of the differences between baseline and time point 3 of log2(BECLIN-1) and log2(pSer258-ATG13) versus all questionnaires. Statistical significance was set at *p* less than 0.05. Statistical analyses were performed using GraphPad Prism 10 for macOS version 10.4.2.

## Results

A total of 109 participants were enrolled in this study between November 2024 and February 2025 (Fig. 2). Following the completion of baseline questionnaires and biospecimen collection, 23 individuals elected not to initiate study drug treatment after consultation with their respective clinical investigators. The remaining 86 participants were initiated on therapy with low-dose rapamycin. Of these, 70 completed the T1 phase (day 36), 55 completed the T2 phase (day 60), and 46 completed the full 90-day study protocol (T3), as shown in the study flowchart.

Overall, the weekly low-dose rapamycin regimen was well tolerated, with minimal adverse events (AEs) reported (Table ##). The most common AEs, transient gastrointestinal symptoms, headache, and insomnia, were primarily observed during the baseline period and declined over time, suggesting adaptation to therapy. Importantly, discontinuation from the study was most commonly attributed to financial barriers as this pilot trial did not cover the cost of the study drug or safety laboratory tests. A secondary reason for discontinuation was a lack of perceived clinical benefit or a clinical decision to initiate a different therapy that may or may not interact with the study protocol.

### Rapamycin provides significant relief from key clinical symptoms in ME/CFS patients as measured by the BAS, SSS, MFI and SF-36. (COMPLETER COHORT)

**Figure 1:**
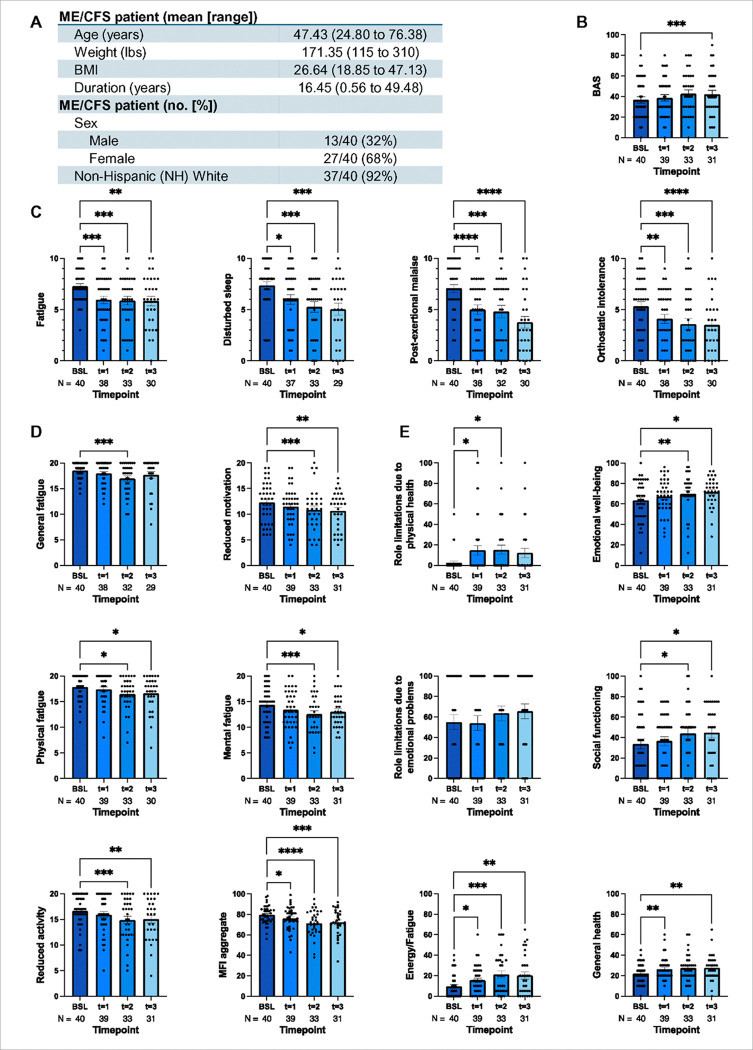
Low-dose rapamycin therapy significantly reduces clinical symptoms of fatigue in ME/CFS subjects. (A) The table summarized general information of the ME/CFS cohort (N=40). Bar charts shown for the (B) Bell Activity Scale (BAS) and (C) Specific Symptom Scale (SSS) inventory of fatigue, disturbed sleep, PEM, and OI. Each point represents the sample of interest. (D) Different measurement criteria of Multidimensional Fatigue Inventory (MFI) including general fatigue, physical fatigue, reduced activity, reduced motivation, mental fatigue, and MFI aggregate were summarized in bar charts with individual scatter points annotated with sample size and timelines at bottom. (E) RAND 36-Item Short Form (SF-36) were analyzed based on role limitations due to physical health, emotional problems, energy/fatigue, emotional well-being, social functioning, and general health. Comparisons are from a mixed-effects model. The choice of statistical analysis and the details were discussed in method and result sections. ^****^: *p* < 0.0001, ^***^: *p* < 0.001, ^**^: *p* < 0.01, *: *p* < 0.05.

The demographic and clinical characteristics of the completer subgroup (N=40), including age, sex, BMI, ethnicity, and disease duration, are summarized in Figure 1A.

#### Bell Activity Scale

Treatment with low-dose rapamycin led to a statistically significant improvement in physical activity levels, as measured by the Bell Activity Scale BAS (Fig. 1B). Mixed model analysis revealed a significant effect of time (*F*_2.6, 87_ = 5.70, ^**^p = 0.002), indicating progressive improvement across the 90-day treatment period. Post-hoc pairwise comparisons using Dunnett’s multiple comparisons test showed a significant 14.99% increase in BAS scores, from a mean of 36.75 at baseline to 42.26 at timepoint 3 (*q*_3, 30_ = 4.83, ^***^p = 0.0001). These findings indicate that clinically meaningful improvements in physical activity emerged gradually over the course of rapamycin therapy, with the most robust effects observed after 90 days of continuous treatment.

#### Specific Symptom Severity Inventory

Low-dose rapamycin treatment resulted in a significant improvement in several core ME/CFS symptoms, as measured by patient-reported outcomes on the Symptom Severity Scale (Fig. 1C). Repeated-measures ANOVA showed statistically significant reductions in fatigue (*F*_2.82, 92.12_ = 9.61, ^****^p < 0.0001), disturbed sleep (*F*_2.42, 77.54_ = 10.68, ^****^p < 0.0001), post-exertional malaise PEM (*F*_2.61, 84.4_ = 17.5, ^****^p < 0.0001), and orthostatic intolerance (OI; *F*_2.55, 83.3_ = 10.9, ^****^p < 0.0001). Post-hoc Dunnett’s tests confirmed significant reduction in fatigue from baseline to time points 1, 2, and 3 (^***^p = 0.0004, ^***^p = 0.0004, and ^***^p = 0.0002, respectively). Similarly, disturbed sleep improved significantly from baseline to each time point (*p = 0.03, ^***^p = 0.0002, and ^***^p = 0.0001), as did PEM (^****^p < 0.0001, ^***^p = 0.0002, and ^****^p < 0.0001) and OI (^**^p = 0.007, ^***^p = 0.001, and ^****^p < 0.0001). These results indicated that rapamycin therapy produced sustained and clinically meaningful improvements in symptoms central to ME/CFS, particularly fatigue, PEM, sleep disturbance, and orthostatic intolerance.

#### Multidimensional Fatigue Inventory

Regarding the MFI (Fig. 1D), patients reported a statistically significant improvement across all fatigue domains: general fatigue (*F*_1.99, 63.8_ = 6.92, ^**^p = 0.002), physical fatigue (*F*_2.51, 82.9_ = 4.01, *p = 0.01), reduced activity (*F*_2.77, 89.5_ = 7.67, ^***^p = 0.0002), reduced motivation (*F*_2.06, 67.9_ = 5.23, ^**^p = 0.007), mental fatigue (*F*_2.56, 84.5_ = 6.92, ^***^p = 0.001), and the aggregate (*F*_2.54, 84.7_ = 13.1, ^****^p < 0.0001). Dunnett’s multiple comparisons test revealed significant improvements from baseline to time point 1 in the aggregate (*q*_3, 38_ = 2.85, *p = 0.02). There were significant improvements from baseline to time point 2 in the general fatigue, physical fatigue, reduced activity, reduced motivation, and mental fatigue domains, and in the aggregate (^***^p = 0.001, *p = 0.02, ^***^p = 0.0007, ^***^p = 0.0004, ^***^p = 0.0009, and ^****^p < 0.0001, respectively), and similar observations were found from baseline to time point 3 in the above domains and aggregates (*p = 0.04, ^**^p = 0.002, ^**^p = 0.005, *p = 0.02, and ^***^p = 0.0002, respectively).

#### RAND 36-Item Short Form

On average, patients generally reported significantly higher scores over time for each SF-36 subscale (Fig. 1E), including physical functioning (*F*_2.8, 92_ = 3.8, *p = 0.02), role limitations due to physical health (*F*_2.32, 77.5_ = 4.17, ^**^p = 0.01), energy and fatigue (*F*_2.72, 90.8_ = 9.94, ^****^p < 0.0001), emotional well-being (*F*_2.53, 84.3_ = 3.78, *p = 0.02), social functioning (*F*_2.43, 81.0_ = 3.92, *p = 0.02), and general health (*F*_2.43, 80.9_ = 4.75, ^**^p = 0.007). However, this was not observed in the role limitations due to emotional problems (*F*_2.66, 88.6_ = 0.84, *p* = 0.46) and bodily pain (*F*_2.5, 82_ = 1.6, *p* = 0.21) subscales. Nonetheless, with the former, this is consistent with the leading theory that ME/CFS is not psychosomatic in etiology^46^. With respect to the aggregate scores, the Physical Component Score (PCS) and the Mental Component Score (MCS), there was no significant improvement in the former (*F*_2.5, 83_ = 2.0, *p* = 0.13), but there was improvement in the latter (*F*_2.9, 95_ = 3.6, *p = 0.02). Post-hoc testing with Dunnett’s test revealed significant improvements from baseline to Time Point 1 in the following subscales: role limitations due to physical health, energy and fatigue, and general health (*p = 0.03, ^**^p = 0.01, and ^**^p = 0.01, respectively). Similar improvements were also found at time point 2 in the role limitations due to physical health, energy and fatigue, emotional well-being, social functioning subscales (*p = 0.02, ^***^p = 0.0002, ^***^p = 0.008, and *p = 0.03), and MCS (*p = 0.03). Significant increases in energy and fatigue, emotional well-being, social functioning, and general health were also found at time point 3 (^**^p = 0.003, *p = 0.03, *p = 0.03, and ^**^p = 0.005).

Collectively, our results suggest that 90 days of low-dose rapamycin treatment significantly improved ME/CFS symptoms, based on the SSS inventory, MFI, and SF-36 scores.

### Patients with a documented history of a viral infection that precedes ME/CFS onset respond better to rapamycin than patients of non-viral onset. (ONSET COHORT)

**Figure 2:**
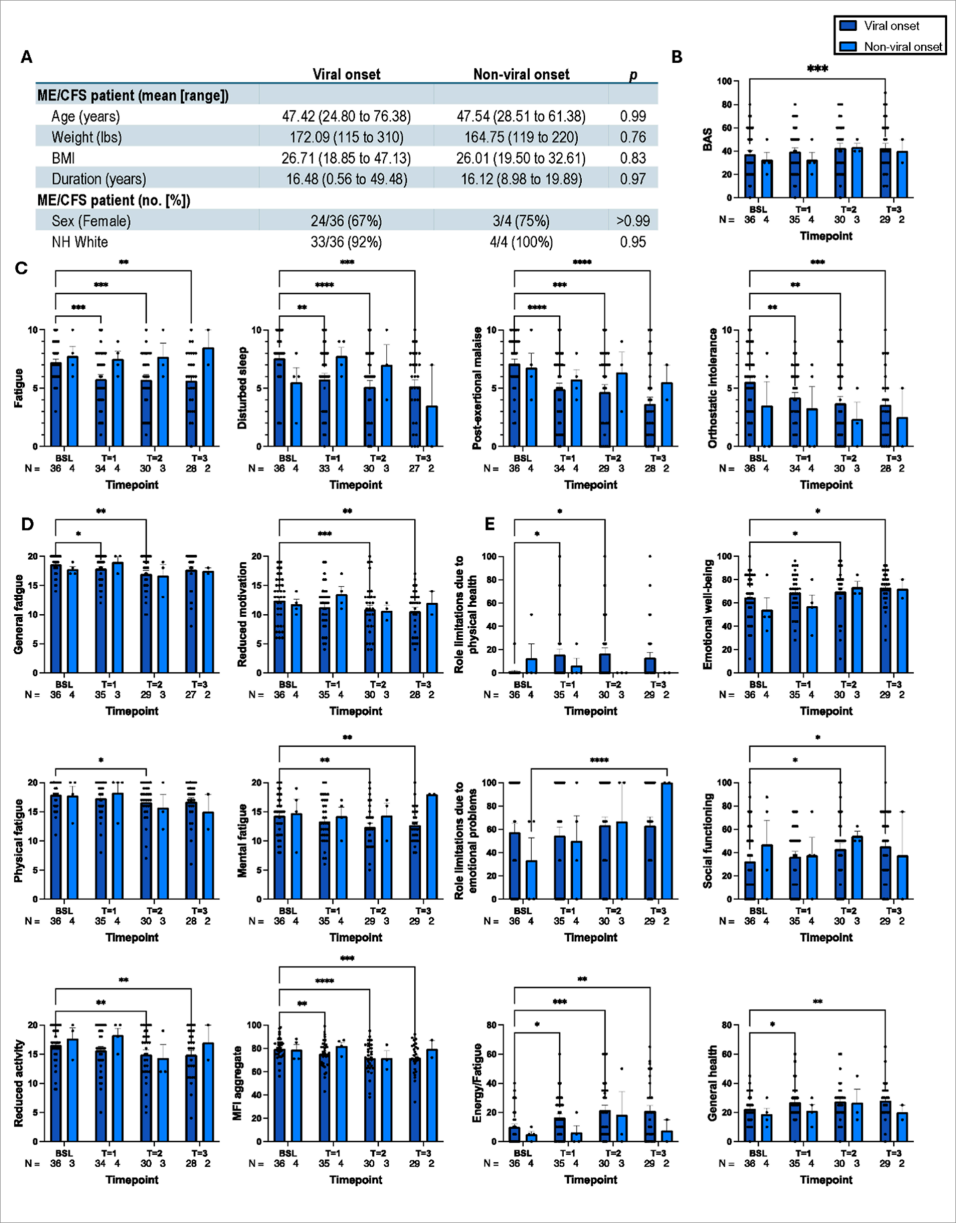
Improvement of fatigue in post-infectious ME/CFS subjects after low dose rapamycin treatment. **(**A) A table represented the details of the cohort (the first column) with mean value and range (the middle columns) and significance (the last column). Fatigue symptoms were evaluated based on (B) BAS, (C) SSS, (D) MFI, and (E) SF-36 measurement criteria. Comparisons are from a stacked mixed-effects model and discussed in detail under result section. ^****^: *p* < 0.0001, ^***^: *p* < 0.001, ^**^: *p* < 0.01, *: *p* < 0.05..

Next, we performed a comparative study of fatigue-related behaviors between ME/CFS subjects with viral and non-viral onsets. A table (Fig. 2A) compares the general health parameters between viral and non-viral onset ME/CFS cases.

On average, post-infectious ME/CFS patients reported more significant improvements across the questionnaires from baseline to timepoint 3 than other ME/CFS patients. In most cases, regarding interaction effects, the differences between the time points were consistent for patients with and without a preceding viral infection. This is true for the BAS (*F*_3, 97_ = 0.76, *p* = 0.52) (Fig. 2B), the fatigue (*F*_3, 95_ = 1.05, *p* = 0.37), PEM (*F*_3, 94_ = 0.61, *p* = 0.61), and orthostatic intolerance (*F*_3, 95_ = 0.46, *p* = 0.71) symptoms in the SSS inventory (Fig. 2C), all domains [general fatigue (*F*_3, 93_ = 0.81, *p* = 0.49), physical fatigue (*F*_3, 96_ = 0.56, *p* = 0.64), reduced activity (*F*_3, 94_ = 2.55, *p* = 0.06), reduced motivation (*F*_3, 96_ = 1.79, *p* = 0.15), and mental fatigue (*F*_3, 96_ = 1.36, *p* = 0.26)] and the aggregate (*F*_3, 97_ = 2.28, *p* = 0.08) for the MFI (Fig. 2D), and all subscales [PF (*F*_3, 97_ = 2.42, *p* = 0.07), RP (*F*_3, 97_ = 0.81, *p* = 0.36), RE (*F*_3, 97_ = 1.94, *p* = 0.13), VT (*F*_3, 97_ = 0.73, *p* = 0.53), MH (*F*_3, 97_ = 0.68, *p* = 0.56), SF (*F*_3, 97_ = 1.14, *p* = 0.34), BP (*F*_3, 97_ = 2.63, *p* = 0.054), and GH (*F*_3, 97_ = 0.91, *p* = 0.44)] and aggregates [PCS (*F*_3, 97_ = 2.02, *p* = 0.12) and MCS (*F*_3, 97_ = 1.68, *p* = 0.18)] for the SF-36 (Fig. 2E).

Post-hoc Dunnett’s multiple comparisons test ascertained that, on average, there were more significant effects from baseline for patients with post-infectious ME/CFS than for other ME/CFS patients. For patients with viral onset, a significant difference was found in BAS (Fig. 2B) from baseline to time point 3 (^***^p = 0.0004) and in SSS fatigue (Fig. 2C) [from baseline to timepoint 1 (^***^p = 0.0002), timepoint 2 (^***^p = 0.0002), and timepoint 3 (^***^p = 0.001)], disturbed sleep [from baseline to timepoint 1 (^***^p = 0.001), timepoint 2 (^****^p < 0.0001), and timepoint 3 (^***^p = 0.0004)], PEM [from baseline to timepoint 1 (^****^p < 0.0001), time point 2 (^***^p = 0.0001), and timepoint 3 (^****^p < 0.0001)], and orthostatic intolerance [from baseline to timepoint 1 (^**^p = 0.007), timepoint 2 (^**^p = 0.002), and timepoint 3 (^***^p = 0.0001)]. With MFI, differences were found in general fatigue (Fig. 2D) [from baseline to timepoint 1 (*p = 0.04) and timepoint 2 (^**^p = 0.002)], physical fatigue from baseline to timepoint 2 (*p = 0.04), reduced activity [from baseline to timepoint 2 (^***^p = 0.001) and timepoint 3 (*p* = 0.002)], reduced motivation [from baseline to timepoint 2 (^***^p = 0.0005) and timepoint 3 (^**^p = 0.003)], mental fatigue [from baseline to timepoint 2 (^***^p = 0.001) and timepoint 3 (^**^p = 0.009)], and aggregate [from baseline to timepoint 1 (^**^p = 0.004), time point 2 (^****^p < 0.0001), and time point 3 (^***^p = 0.0002)]. Differences were found in the SF-36 PF (Fig. 2E) from baseline to timepoint 3 (*p = 0.04), RP [from baseline to timepoint 1 (*p = 0.02) and time point 2 (*p = 0.02)], VT [from baseline to timepoint 1 (*p = 0.01), time point 2 (^***^p = 0.0002), and timepoint 3 (^**^p = 0.004)], MH [from baseline to timepoint 2 (*p = 0.03) and timepoint 3 (*p = 0.047)], SF [from baseline to timepoint 2 (*p = 0.04) and timepoint 3 (*p = 0.03)], and GH subscales [from baseline to timepoint 1 (*p = 0.02) and timepoint 3 (^**^p = 0.003)]. No significant differences were found with Dunnett’s test for non-viral onset patients, with some instruments not having a large enough sample size to properly compare the groups.

Overall, our results show that post-infectious ME/CFS patients had significantly better responses to rapamycin than non-viral ME/CFS patients based on the BAS score, SSS inventory, MFI, and SF-36 criteria.

### Observed effects vary in magnitude depending on responder status: RESPONDER SPECTRUM COHORT

**Figure 3:**
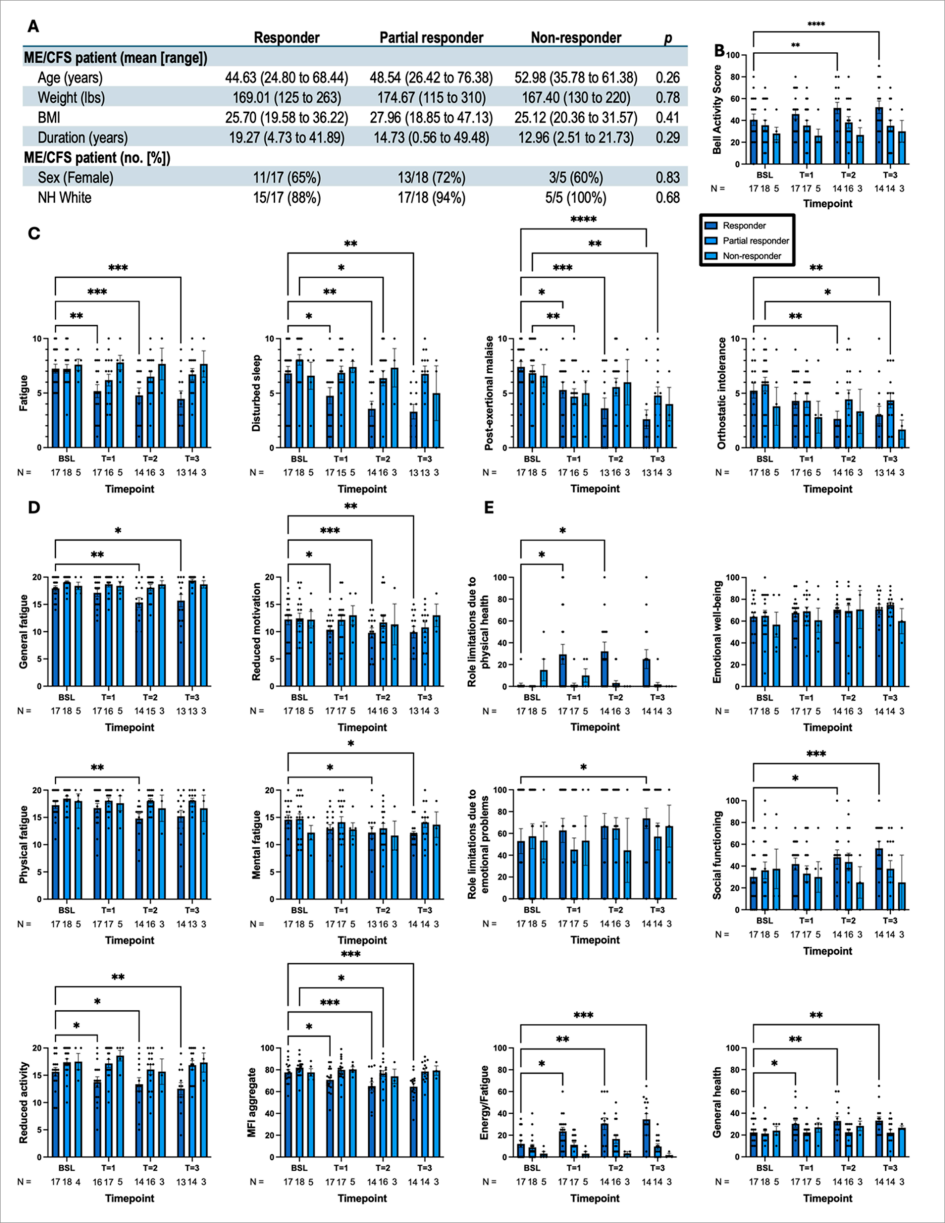
Analysis the effect of rapamycin on fatigue-related symptoms in ME/CFS subjects stratified based on responder, partial responder, and non-responder groups. **(**A) A table presented the basic information of the cohort based on responder (the first column), partial responder (the middle column), and the non-responder (the last column) groups. Fatigue symptoms were evaluated based on (B) BAS, (C) SSS, (D) MFI, and (E) SF-36 measurement criteria. Comparisons are from a stacked mixed-effects model and discussed in detail under result section. ^****^: *p* < 0.0001, ^***^: *p* < 0.001, ^**^: *p* < 0.01, *: *p* < 0.05..

The heterogeneity of patients with ME/CFS indicates a spectrum of responses to any given therapy. Given that we found a spectrum of responders in our overall completer cohort, we next aimed to track the individual responders, partial responders, and non-responders in this study, as shown in (Fig. 3A). The protocol described in Methods section describes how responders were stratified. Unlike the viral subset analysis, several surveys revealed interaction effects in which, on average, responders had greater and more significant differences over time than partial responders and non-responders. For example, with BAS (Fig. 3B), compared to the baseline, responders showed more significant improvements than the other subsets (*F*_6, 94_ = 3.11, ^**^p = 0.01). Further testing with Dunnett’s method showed significant improvements among responders in the BAS from baseline to time point 2 (^**^p = 0.006) and time point 3 (^****^p < 0.0001).

For the SSS inventory (Fig. 3C), only responders showed significant decreases in select ME/CFS symptoms. For responders, significant (*F*_6, 94_ = 3.11, ^**^p = 0.004) improvements were observed from baseline to all time points (^**^p = 0.002, ^***^p = 0.0004, and ^***^p = 0.0009, respectively). For disturbed sleep symptoms, significant (*F*_6, 92_ = 3.51, *p = 0.04) improvements were observed for responders and partial responders. Regarding the former, symptoms improved from baseline to all time points (*p = 0.04, ^***^p = 0.008, and ^**^p = 0.004), and for the latter, improvements were observed from baseline to time point 2 (*p = 0.03). Similarly, significant (*F*_6, 91_ = 3.15, ^**^p = 0.008) improvements in PEM were seen in responders from baseline to all time points (*p = 0.03, ^***^p = 0.0002, and ^****^p < 0.0001) and in partial responders from baseline to time points 1 (^**^p = 0.004) and 3 (^**^p = 0.002). No significant effects were found on orthostatic intolerance symptoms when stratified by responders.

With MFI (Fig. 3D), only responders were reported to have significant improvements over time from multiple comparison testing. These effects were observed in the general fatigue (*F*_6, 90_ = 3.43, ^**^p = 0.004) and reduced activity (*F*_6, 91_ = 2.30, *p = 0.04) domains, and MFI aggregates (*F*_6, 94_ = 3.40, ^**^p = 0.005). Dunnett’s multiple comparisons test revealed a significant decrease in general fatigue from baseline to time point 2 (^**^p = 0.003) and time point 3 (*p = 0.04), reduced activity from baseline to all time points (*p = 0.04, *p = 0.01, and ^**^p = 0.001, respectively), and aggregation from baseline to all time points (*p = 0.01, ^***^p = 0.0002, and ^***^p = 0.0005, respectively). Exceptionally, partial responders showed improvement (*p = 0.02) in MFI aggregate from baseline to time point 2.

With respect to SF-36 (Fig. 3E), significant interaction effects were observed; only responders had significant effects, with PF (*F*_6, 94_ = 3.88, ^**^p = 0.002), RP (*F*_6, 94_ = 3.31, ^**^p = 0.005), VT (*F*_6, 94_ = 4.77, ^***^p = 0.0003), SF (*F*_6, 94_ = 3.51, ^**^p = 0.004), and PCS aggregate (*F*_6, 94_ = 3.25, ^**^p = 0.006). Post-hoc testing with Dunnett’s method revealed significant differences in PF from baseline to timepoint 2 (*p = 0.02) and timepoint 3 (^**^p = 0.003), in RP from baseline to timepoint 1 (*p = 0.02) and timepoint 2 (*p = 0.01), in VT from baseline to all time points (*p = 0.02, ^**^p = 0.002, and ^***^p = 0.0002, respectively), in SF from baseline to timepoint 2 (*p = 0.04) and time point 3 (^***^p = 0.0006), and in PCS from baseline to timepoint 2 (*p = 0.01) and timepoint 3 (*p = 0.02).

In summary, similar results were observed using responder subsets, similar to our viral subset analysis; however, one additional group, partial responders, had slightly more significant effects than non-responders and slightly fewer effects than responders.

### Effect of rapamycin on the improvement of autophagy in trial participants:

#### Analyzing autophagy in completer cohort

As an mTOR inhibitor, rapamycin is expected to improve autophagy flux^47^ in ME/CFS patients, and increasing levels of serum BECLIN-1 are considered reliable markers for the overall improvement of autophagy flux^48,49^.

**Figure 4:**
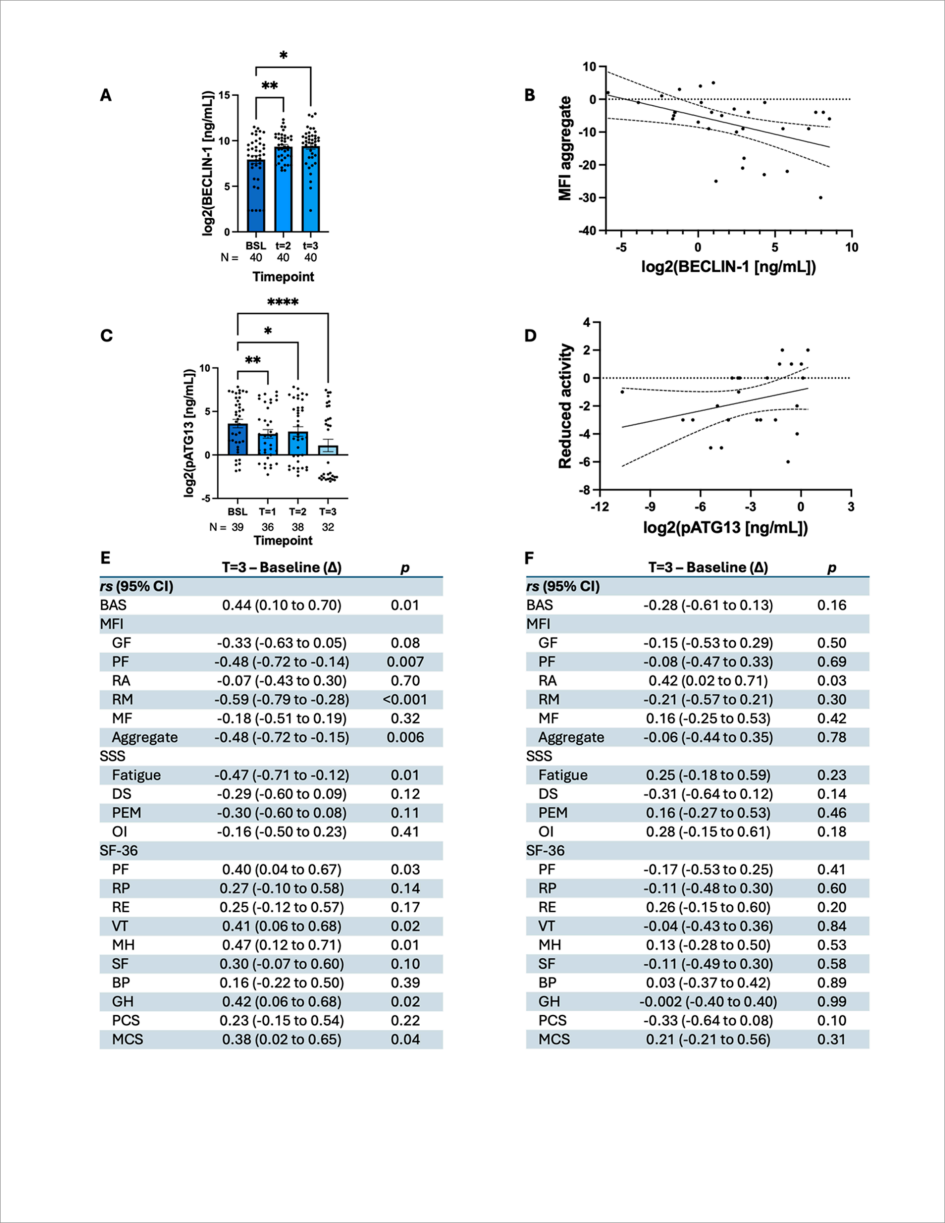
Low-dose rapamycin therapy significantly increased autophagy marker BECLIN-1 and reduced pSer258-ATG13 and in ME/CFS subjects. (A) BECLIN-1 ELISA was performed in the plasma samples, converted to a logarithmic (base-2) scale, and then plotted as a dotted bar chart for different milestones. Sample size was included at the bottom of each bar. (B) The correlation curve of the MFI aggregate score vs BECLIN-1 represents a significant (^**^p = 0.006) relationship. (C) pSer258-ATG13 ELISA was performed in plasma samples, converted to a logarithmic (base-2) scale, and plotted as a bar chart. (D) A significant (*p = 0.03) correlation between MFI reduced activity (RA) vs pSer258-ATG13 was shown. Spearman coefficients (*rs*) for (E) BECLIN-1 and (F) pSer258-ATG13 for each questionnaire (BAS, SSS, MFI, and SF-36) are summarized in a table. Comparisons are from an (A) RM ANOVA or (C) mixed-effects model. ^****^: *p* < 0.0001, ^***^: *p* < 0.001, ^**^: *p* < 0.01, *: *p* < 0.05..

Interestingly, we observed a significant upregulation of BECLIN-1 (*F*_1.5, 57_ = 6.5, ^**^p = 0.007) in all ME/CFS cases (N=40) (Fig. 4A). Dunnett’s multiple comparisons test further revealed significant increases of 1.4-and 1.5-fold from baseline to time points 2 (*q*_2, 39_ = 3.0, ^**^p = 0.01) and 3 (*q*_2, 39_ = 2.6, *p = 0.02), respectively.

Our previous study [10] identified elevated serum concentrations of multiple phosphorylated ATG13 species as a potential pathogenic mechanism of ME/CFS. However, the antibody used (targeting Ser344) lacked specificity for detecting the mTOR-dependent phosphorylation of ATG13 in human samples, and no commercially available antibody was available. To accurately assess this, we developed a novel pSer258-ATG13 antibody to optimize the detection of pSer258-ATG13 by ELISA (**Supplementary fig. 3**) We observed a significant reduction in circulating pSer258-ATG13 concentrations following initiation of low-dose rapamycin therapy (F_1·59_, 54.2 = 22.1, ^****^p < 0.0001; Fig. 4B). Post-hoc analyses demonstrated a consistent decrease in pSer258-ATG13 levels from baseline across all time points: a one-fold reduction at T1 (^**^p = 0.005), a 0.8-fold reduction at T2 (*p = 0.02), and a more than two-fold reduction by T3 (^****^p < 0.0001). These findings suggest that low-dose rapamycin effectively suppresses mTORC1-mediated phosphorylation of ATG13 over time in patients with ME/CFS.

Next, we performed a series of correlation analyses to evaluate the relationship between the changes in autophagy markers and fatigue-related symptom scores (Fig. 4C–F). Increases in BECLIN-1 levels from baseline to T3 were positively correlated with improvements in several clinical outcome measures, including the BAS (r〿 = 0.44, ^**^p = 0.01) and multiple subscales of the SF-36: physical functioning (PF; r〿 = 0.40, *p = 0.03), energy and fatigue (VT; r〿 = 0.41, *p = 0.02), emotional well-being (MH; r〿 = 0.47, ^**^p = 0.01), general health (GH; r〿 = 0.42, *p = 0.02), and Mental Component Summary (MCS; r〿 = 0.38, *p = 0.04).

Conversely, BECLIN-1 levels were negatively correlated with several subdomains of the MFI including physical fatigue (r〿 = −0.48, ^**^p = 0.007), reduced motivation (r〿 = −0.59, ^****^p < 0.0001), and the MFI aggregate score (r〿 = −0.48, ^**^p = 0.006), as well as the fatigue symptom domain of the Symptoms Severity Scale (SSS; r〿 = −0.47, ^**^p = 0.01). Linear regression analysis confirmed a strong inverse relationship between the BECLIN-1 levels and MFI aggregate scores (Fig. 4D).

In contrast, when evaluating pSer258-ATG13, we observed a significant positive correlation between its reduction and improvement in the MFI Reduced Activity domain (*p = 0.03; Fig. 4E). However, no significant correlations were observed between pSer258-ATG13 and the other MFI domains.

#### Analyzing autophagy in post-infectious ME/CFS subjects:

Next, we evaluated the effect of rapamycin on autophagy flux in a subset of post-infectious ME/CFS participants, specifically those with a documented viral illness preceding the onset of ME/CFS symptoms.

**Figure 5:**
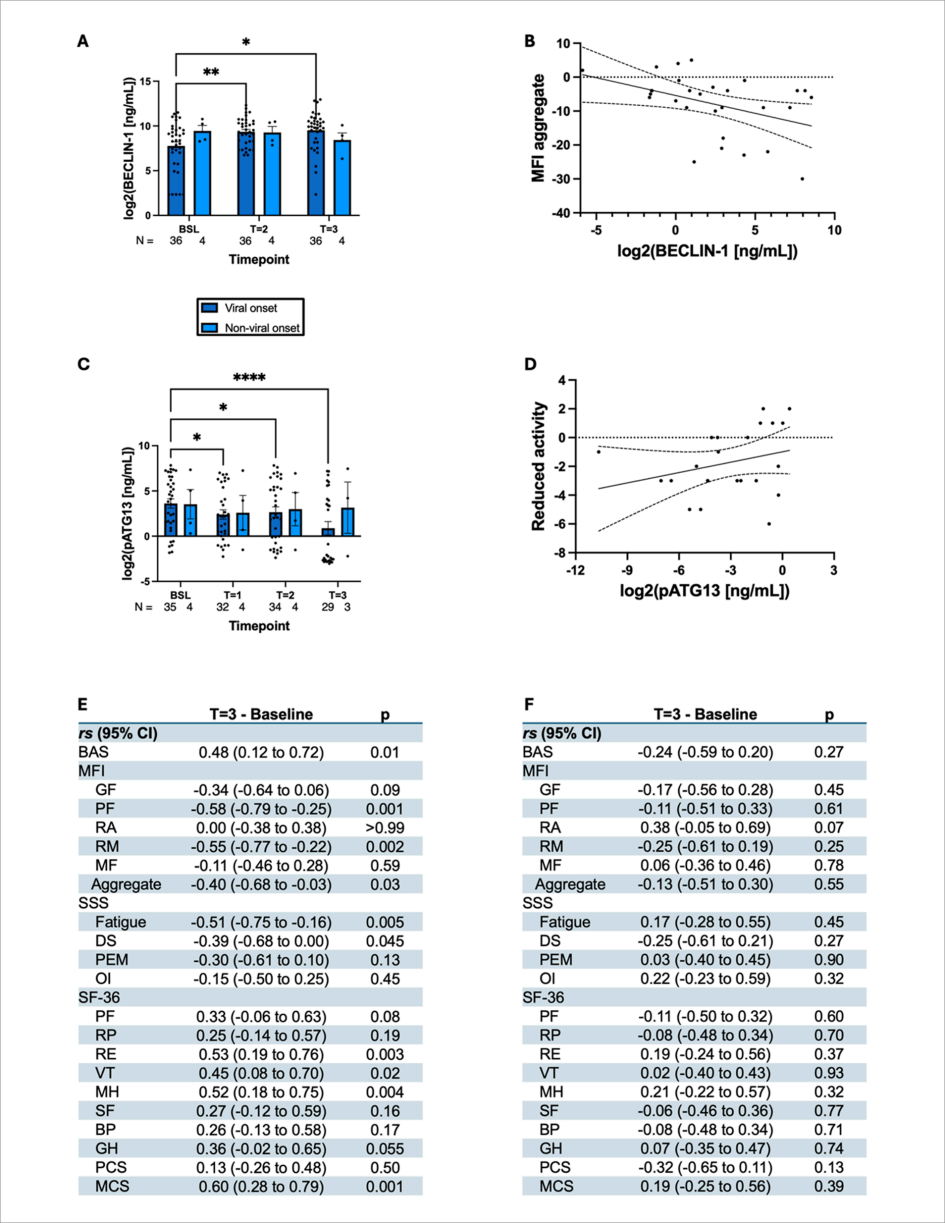
Low-dose rapamycin therapy upregulated autophagy marker BECLIN-1 and suppressed pSer258-ATG13 in ME/CFS subjects stratified by history of viral onset. **(**A) The plasm level of BECLIN-1 was measured by ELISA and plotted in a log scale as a dotted bar chart for each milestone. Each bar was annotated with sample size underneath. (B) MFI aggregate. vs BECLIN-1 curve displayed a significant (*p = 0.03) relationship. (C) pSer258-ATG13 ELISA was performed and plotted in a log scale as a dotted bar chart for each milestone. Each bar was annotated with sample size underneath. (D) The relationship between MFI RA vs pSer258-ATG13 was demonstrated. (E) Spearman coefficients for BECLIN-1 with each questionnaire. (F) Spearman coefficients for pSer258-ATG13 for each of the measurement criteria. B, D, E-F = for patients with history of viral onset only. A, C: Comparisons are from a (A) two-way ANOVA or (C) stacked mixed-effects model. ^****^: *p* < 0.0001, ^***^: *p* < 0.001, ^**^: *p* < 0.01, *: *p* < 0.05..

Interestingly, post-hoc testing with Dunnett’s method revealed that patients with viral onset ME/CFS had significant increases in BECLIN-1 (Fig. 5A) from baseline to time points 2 (^**^p = 0.007) and 3 (^**^p = 0.01). In contrast, patients with non-viral onsets were found to have no statistically significant increase in BECLIN-1 concentrations from baseline to time point 2 (*p* = 0.98) or time point 3 (*p* = 0.69). One caveat to this analysis is the much smaller sample size for patients with non-viral onset ME/CFS (N=4) compared to viral onset (N=36), which may not be truly representative of the effect size.

Similarly, Dunnett’s test showed that patients who had a history of viral onset prior to ME/CFS diagnosis had significantly decreased Serine258 pATG13 levels (Fig. 5B) than those who did not. In patients with viral onset, these improvements were one-fold at time point 1 (^**^p = 0.01), 0.84-fold at time point 2 (*p = 0.03), and 2.7-fold at time point 3 (^****^p < 0.0001).

Among participants with a history of viral-onset ME/CFS, changes in BECLIN-1 levels from baseline to time point 3 were positively correlated with improvements in several clinical outcomes (Fig. 5C–F). Specifically, BECLIN-1 increases were significantly associated with improvements in the BAS (r〿 = 0.48, ^**^p = 0.01), as well as multiple domains of the SF-36: RE (r〿 = 0.53, ^**^p = 0.003), VT (r〿 = 0.45, *p = 0.02), MH (r〿 = 0.52, ^**^p = 0.004), and the MCS (r〿 = 0.60, ^***^p = 0.001).

In contrast, BECLIN-1 levels were negatively correlated with symptom severity as measured by the MFI: physical fatigue (r〿 = −0.58, ^***^p = 0.001), reduced motivation (r〿 = −0.55, ^**^p = 0.002), and the MFI aggregate score (r〿 = −0.40, *p = 0.03) (Fig. 5D). Similarly, significant negative correlations were observed between the fatigue (r〿 = −0.51, ^**^p = 0.005) and disturbed sleep (r〿 = −0.39, *p = 0.045) domains of the SSS.

Owing to the limited sample size, correlation analyses could not be conducted in the subgroup of ME/CFS patients without a history of viral onset. Furthermore, no significant correlations were observed between pSer258-ATG13 levels and any MFI subdomain in the viral-onset group, including reduced activity (*p* = 0.07) (Fig. 5E–F).

#### Analyzing autophagy in responder, partial responder, and non-responder ME/CFS subjects:

Next, we analyzed the improvement of autophagy flux (BECLIN-1, **Supplementary Fig. 4A**) and further investigated the correlation between MFI and autophagy markers in responders, partial responders, and non-responders. No significant interaction effects were found for BECLIN-1 concentrations, but Dunnett’s test revealed significant increases for responders only from baseline to time points 2 and 3 of 2.6- and three-fold, respectively (*p = 0.01, *p = 0.01, respectively). There were no significant differences between the partial responders and non-responders.

Likewise, there were no significant interaction effects vis-à-vis pSer258-ATG13 concentration (**Supplementary Fig. 4B**). Multiple comparison testing was still performed, in which responders had a 1.5-fold decrease (^**^p = 0.001) in pSer258-ATG13 from baseline to timepoint 1. Responders and partial responders both showed a three-fold and 2.3-fold (, respectively) decrease from baseline to time point 3 (^**^p = 0.001 and *p = 0.02, respectively).

With respect to the responder subsets, no significant correlation was observed between some questionnaires and BECLIN-1 (**Supplementary Fig. 4C& 4E**) and pSer258-ATG13 (**Supplementary Fig. 4D and 4F**) levels. The exceptions were only partial responders, and they included BECLIN-1 versus MFI reduced motivation (*r*_*s*_ = −0.56, *p = 0.04), MFI mental fatigue (albeit in an unexpectedly different direction; *r*_*s*_= 0.61, *p = 0.02), pSer258-ATG13 versus the SSS disturbed sleep (unexpectedly in a different direction; *r*_*s*_ = −0.63, *p = 0.04), SSS orthostatic intolerance (expected direction: *r*_*s*_ = 0.71, *p = 0.01), and SF-36 MCS (expected direction; *r*_*s*_ = 0.59, *p = 0.049). However, we expect other correlations to exist for a larger sample size.

#### Low-dose rapamycin does not affect clinical safety lab results.

mTOR is a central regulator of key cellular processes including metabolism and autophagy. Rapamycin, a selective mTOR inhibitor, is hypothesized to enhance autophagic activity by promoting sequestration of phosphorylated ATG13. However, mTOR inhibition may also suppress essential metabolic functions such as lipolysis and protein catabolism. Prior studies in both animal models and humans have shown that high-dose daily rapamycin administration can lead to adverse metabolic effects, including hypercholesterolemia, anemia, thrombocytopenia, and insulin resistance. In contrast, using our low-dose weekly administration protocol, we observed no adverse changes in safety laboratory parameters in our cohort from baseline through time point 3 **(Supplementary Figs. 5A–6B)**. This absence of laboratory abnormalities, combined with the low incidence of reported adverse events, highlights the favorable safety profile of weekly low-dose rapamycin in patients with ME/CFS.

Taken together, our pilot study demonstrated that low-dose rapamycin (6 mg/week) significantly ameliorated the clinical symptoms of fatigue and was well-tolerated in this relatively large ME/CFS cohort. Importantly, these findings support a mechanistic role of mTOR suppression in the restoration of autophagy. Rapamycin treatment is associated with a marked reduction in pSer258-ATG13, a key indicator of impaired autophagy. This reduction was correlated with an increase in BECLIN-1 expression, consistent with enhanced autophagy flux.

#### A proposed machine learning model to predict responder subtype for future analyses:

Using the procedure described in the Methods section, we were able to train a random forest machine learning model in which participants could be stratified based on previously verified and novel MCIDs for the different questionnaires. Missing data were initially imputed using KNN with the nearest neighbors set to 2. The data were 80–20 split such that 80% of the data were used for training, and 20% were used for testing. The initial accuracy of the random forest model is 0.75. Hyperparameter tuning via 5-fold cross-validation GridSearch determined the most efficient parameter for KNN imputation to be 2, so no changes were made for preprocessing. The optimal parameters for the random forest were determined to be a max_depth of two, max_features of sqrt, min_samples_leaf of one, min_samples_split of two, and n_estimators of 200. We found that the depth of 2 was not sufficient to be a subset into three groups, so we increased the max_depth to 3. The accuracy was found to be 0.78 using these updated parameters. One of the trees in the random forest is shown in **Supplementary Fig. 7**. The out-of-bag (OOB) score was found to be 0.78, and a check for overfitting found the training accuracy to be 0.94.

A feature importance analysis was also performed. The model determined that the three predictors with the highest fidelity for determining the responder subset were the BAS (0.18), SF-36 PCS aggregate (0.15), and SF-36 energy/fatigue subscale (0.13); the least important predictor was the MFI reduced motivation domain (0.02). A confusion matrix (**Supplementary Fig. 8)** was plotted in which the model placed responders with the highest accuracy. The model tended to be both over- and under-predicted partial responders. Finally, an AUC ROC curve (**Supplementary Fig. 9**) was plotted to provide more context for the confusion matrix. The non-responder subset had the highest AUC of 1.00, followed by the responder subset, with an AUC of 0.92. The lowest AUC 0.71 was for the partial responder subset, which often provided more false positives. We have demonstrated that the model has high fidelity for the binary separation of responders and non-responders, and future directions with more edge cases would improve the accuracy of the model in the partition of partial responders.

## Discussion

Myalgic encephalomyelitis/chronic fatigue syndrome (ME/CFS) is a debilitating, multisystem chronic illness with no approved treatments or objective diagnostic biomarkers. Our prior research in both human subjects and a mechanistically informed animal model identified chronic mTOR activation and subsequent autophagy impairment as potential drivers of ME/CFS pathogenesis, particularly in relation to post-exertional malaise (PEM), a hallmark symptom of the disease. Leveraging this mechanistic insight, the present study assessed the clinical efficacy of weekly low-dose rapamycin in improving disease-defining symptoms in patients with ME/CFS. Importantly, we also developed two blood-based molecular assays targeting BECLIN-1 and pSer258-ATG13 as objective biomarkers of autophagy function, which may facilitate future stratification of responders and guide precision therapeutic approaches in this highly heterogeneous population.

Rapamycin was well tolerated in this cohort, with a low incidence of adverse events, most of which were transient and nonserious. No significant changes were observed in safety laboratory parameters across the study duration, and there was no evidence of increased susceptibility to infections, addressing concerns regarding immunosuppression at this dosing schedule. These findings support the safety of once-weekly low-dose rapamycin in ME/CFS and justify further investigation in larger controlled trials.

### Rapamycin recovers ME/CFS patients (N=40) from physical and mental fatigue symptoms.

Several lines of this manuscript demonstrate that rapamycin treatment effectively ameliorated fatigue-related symptoms in ME/CFS subjects. *First,* BAS analysis revealed a strong and significant improvement in daily physical activity in all participants from the BSL to T3 stages. *Second*, symptom analysis from the SSS inventory indicated a strong and significant recovery in overall fatigue, disturbed sleep, post-exertional malaise, and orthostatic intolerance among ME/CFS patients. *Third*, evaluations of different fatigue parameters of MFI also revealed a significant restoration of health deficits, such as general fatigue, reduced motivation, physical fatigue, mental fatigue, and reduced activity. Finally, the SF-36 score analysis revealed a strong recovery of social functioning and physical and emotional health. Our analysis was unbiased, based on age, sex, body weight, and disease duration. The effect of rapamycin on the overall metabolism of sugars, lipids, and cholesterol was monitored in all patients during each milestone. However, no significant differences were observed.

### Rapamycin may display a strong recovery of fatigue symptoms in post-infectious ME/CFS subjects.

Interestingly, while comparing the effect of rapamycin between post-infectious and no-infection-onset ME/CFS patients, our study identified a significant recovery of fatigue symptoms in the post-infectious group. Based on our analyses of clinical symptoms from the BAS, SSS inventory, MFI, and SF-36, we observed a strong recovery of post-infectious ME/CFS subjects from physical and mental fatigue. Confounding errors did not influence our analysis because of the age, sex, BMI, and disease duration of the post-infectious cohort. Our postinfectious cohort had a median of 16.48 years of disease duration following their viral onset. These patients had a documented history of EBV infection. The non-viral onset group had a disease duration of 16.12 years.

### Rapamycin exhibits a significant improvement of autophagy flux in ME/CFS subjects.

Rapamycin inhibits mTOR and is therefore expected to repair the mTOR-driven inhibition of autophagy. To investigate the effect of rapamycin on the improvement of the overall autophagy flux, we performed a quantitative ELISA using the pan-autophagy marker BECLIN-1. Interestingly, rapamycin significantly increased the plasma levels of BECLIN-1 at the T2 and T3 stages compared to BSL, suggesting that rapamycin may improve the overall autophagy flux in ME/CFS subjects. To better understand the effect of rapamycin on autophagy, we categorized the cohort into three different groups based on the recovery score. The “responder” group (N=17) with the most significant fatigue recovery based on MFI and SF-36 evaluations, displayed the strongest response of BECLIN-1 upregulation from BSL to T2 to T3 stages. The “partial responder” (N=18) group and “non-responder” (N=5) groups did not show any significant upregulation in BECLIN-1 levels. Interestingly, while evaluating the plasma levels of BECLIN-1 in the viral-onset (N=36 ME/CFS cohort), we observed a robust upregulation of BECLIN-1 from BSL to T2 and T3 stages, suggesting that long-term treatment with rapamycin improves overall autophagy in post-infectious ME/CFS subjects. Next, we studied whether the upregulation of BECLIN-1 was correlated with the alleviation of fatigue symptoms. Our correlation analysis suggests a significant correlation between BECLIN-1 upregulation and the alleviation of symptoms, as measured by the MFI total score and two key sub-scores provided by the instrument, including physical fatigue and reduced motivation. Similarly, the correlation was also very strong in the viral-onset group, suggesting that rapamycin treatment could be an effective intervention in a subset of patients with documented beta herpes virus onset and/or reactivation syndrome.

ATG13 is considered a direct target of mTOR. Upon phosphorylation at its serine 258 residue, ATG13 becomes inactivated and aborts the initiation complex of autophagosome formation, resulting in the impairment of cellular autophagy. Our recent animal model study also showed that the chronic activation of mTOR inhibits ATG13 in inducing ME/CFS-like post-exertional malaise. To determine the clinical relevance of pSer258-ATG13 inactivation in ME/CFS subjects, we developed a Serine258P-specific ATG13 antibody that was used in a highly sensitive indirect ELISA. Interestingly, our pSer258-ATG13 ELISA study demonstrated a significant downregulation of pSer258-ATG13 from the BSL to the T1, T2, and T3 stages in N=40 ME/CFS patients. While correlating the pSer258-ATG13 level with clinical symptoms of fatigue, it was found that the downregulation of the pSer258-ATG13 level was positively correlated with the alleviation of the reduced activity domain in the MFI. Similarly, we observed strong downregulation in the plasma levels of pSer258-ATG13 in the rapamycin-treated post-infectious ME/CFS cohort. The downregulation was significant from BSL to all three stages, with the maximum reduction at the T3 stage. However, no significant correlation was observed between pSer258-ATG13 downregulation and alleviated fatigue symptoms in the viral-onset ME/CFS group, mainly because of a dramatic decrease in pSer258-ATG13 at the T3 stage.

### Significance, limitations, challenges, and future directions.

This study represents one of the first biomarker-targeted clinical trials of ME/CFS. To date, no definitive molecular mechanism has been established for the pathogenesis of ME/CFS, and no prior treatment trials have focused on mechanistic targets. Our findings provide the first clinical evidence linking mTOR activation and impaired autophagy to the ME/CFS pathophysiology. Notably, the observed clinical improvement in fatigue was paralleled by molecular evidence of enhanced autophagy, including decreased pSer258-ATG13 and increased BECLIN-1 expression. This trial provides a critically important opportunity to study a subgroup of patients that can be stratified before enrollment based on a convenient molecular test that is likely to respond to a very safe, affordable, and FDA-approved agent.

However, this study had several limitations. Most notably, the absence of a placebo control group limited the ability to definitively attribute clinical improvements to the intervention. Functional outcomes were assessed solely through self-reported questionnaires without objective performance-based measures. Despite these limitations, the trial was designed to test a biomarker-driven hypothesis, namely that rapamycin modulates mTOR-mediated autophagy disruption, and each participant served as their own control. This within-subject design strengthens the internal validity of our findings and supports the potential use of autophagy-related biomarkers, such as BECLIN-1 and pSer258-ATG13, as future inclusion and exclusion criteria in randomized, placebo-controlled trials. Therefore, while not a controlled study, it is important to note that identifying subsets of patients using objective biomarkers like those described in this study is critical for the success of larger controlled trials, given the known heterogeneity of this patient population.

Although a substantial number of participants discontinued the study before the final endpoint, we attribute this primarily to the pilot nature of the trial and limited funding, which prevented us from covering drug and laboratory costs for participants. A secondary reason for dropout was the lack of perceived clinical benefit or the initiation of alternative therapies that were incompatible with the study protocol. We believe that a fully funded, double-blind, placebo-controlled trial with adequate statistical power and cost coverage will be critical to addressing these limitations and validating our preliminary findings.

Another limitation of this study is the absence of a standardized generic formulation of rapamycin. Prior research has demonstrated considerable variability in the bioavailability of different generic sirolimus preparations, which may influence therapeutic response. Future studies should prioritize the use of a single, well-characterized formulation, whether compounded or commercially available, and incorporate pharmacogenetic profiling of drug-metabolizing enzymes, including CYP3A and other cytochrome P450 isoforms. Additionally, measuring peak and trough sirolimus levels is essential to identify fast versus slow metabolizers, enabling individualized dosing strategies to optimize therapeutic efficacy.

While trends in symptom improvement correlated with significant and positive changes in autophagy markers, statistical significance was not achieved in some analyses, likely because of the small sample size. Notably, subgroup analysis in patients with non-viral ME/CFS onset was limited by low enrollment, reducing the power to detect changes in pSer258-ATG13 expression. Additionally, maintaining participant engagement throughout the trial posed logistical challenges.

To address these challenges, an enhanced trial phase is underway, incorporating a larger cohort and improved infrastructure to address the aforementioned limitations. This includes collaboration with key industry partners versed in large, decentralized, double-blinded controlled studies to support centralized drug distribution, enhanced biospecimen collection, inclusion of wearable devices to objectively monitor clinical endpoints, and rapid metabolic and biomarker testing aimed at enhancing clinical effectiveness and identifying new subgroups of responders. These enhancements are expected to improve the trial efficiency, data quality, and clinical impact.

## Figures and Tables

**Table 1.** Treatment stops at each timepoint. Provided were the reasons why participants decided to stop rapamycin therapy.

	RAMP Period	T1 to T2	T2 to T3
**Adverse Events/Reason**			
Diarrhea/GI complaints	5	2	0
Insomnia	3	3	0
Headache	0	1	0
No change in symptoms	0	8	9
Financially unable	8	2	0
**Total**	16	15	9

## Data Availability

There are no electronic datasheets associated with this study. No data were available in the electronic repository.

## Highlights

- The first decentralized biomaker-directed clinical trial of rapamycin, an mTOR inhibitor, was used to treat ME/CFS.
- The correlation between autophagy markers such as BECLIN-1 and pSer258-ATG13 and hallmark symptoms of ME/CFS patients with post-exertional malaise.
- Validation of phosphorylated ATG13 (Ser258) in plasma samples and exploration of its correlation with key disease-defining symptoms in ME/CFS patients.

## Abbreviations

ME/CFS: Myalgic Encephalomyelitis/Chronic Fatigue Syndrome; rapamycin
mTOR: the mammalian target of Rapamycin; BECLIN-1
ATG13: autophagy-related gene13-encoded protein
pSer258-ATG13: serine-258 phosphorylated ATG13
BAS: Bell Activity Scale
SSS: Specific Symptom Severity
PEM: post-exertional malaise
OI: orthostatic intolerance
MFI: Multidimensional Fatigue Inventory
SF-36: RAND 36-Item Short Form Survey
PF: SF-36 physical functioning subscale
RP: SF-36 role limitations due to physical health subscale
RE: SF-36 role limitations due to emotional problems subscale
VT: SF-36 energy/fatigue subscale
MH: SF-36 emotional well-being subscale
SF: SF-36 social functioning subscale
BP: SF-36 bodily pain subscale
GH: SF-36 general health subscale
PCS: SF-36 Physical Component Score aggregate
MCS: SF-36 Mental Component Score aggregate
